# Are Shoulders with A Reverse Shoulder Prosthesis Strong Enough? A Pilot Study

**DOI:** 10.1007/s11999-012-2277-8

**Published:** 2012-02-11

**Authors:** Tjarco D. W. Alta, H. E. J. Veeger, Thomas W. J. Janssen, W. Jaap Willems

**Affiliations:** 1 Department of Orthopaedic Surgery and Traumatology, Onze Lieve Vrouwe Gasthuis, P.O. Box 95500, 1090 HM Amsterdam, The Netherlands; 2 Research Institute MOVE, Faculty of Human Movement Sciences, VU University, Amsterdam, The Netherlands; 3 Section Biomechatronics and Biorobotics, Faculty of Mechanical, Maritime and Materials Engineering, Delft University of Technology, Delft, The Netherlands; 4 Duyvensz-Nagel Research Laboratory, Reade, Centre for Rehabilitation and Rheumatology, Amsterdam, The Netherlands

## Abstract

**Background:**

It has been suggested that limited active ROM of reverse shoulder prostheses relates to lack of strength. However, the postoperative strength has not been quantified.

**Questions/purposes:**

We therefore measured joint torques in patients with reverse shoulder prostheses and correlated torques with functional scores.

**Methods:**

We recruited 33 patients (age, 72 ± 8 years) with a reverse prosthesis (37 shoulders, 21 primary and 16 revisions). We obtained Constant-Murley, DASH, and Simple Shoulder Test ([D]SST) scores, and performed two isokinetic protocols (abduction/adduction and external/internal rotation) at 60° per second. Minimum followup was 4 months (average, 23 months; range, 4–63 months).

**Results:**

Twenty-three patients (24 shoulders; 13 primaries, 11 revisions) were able to perform at least one of the defined tasks. Mean abduction and adduction torques were 15 Nm ± 7 Nm and 16 Nm ± 10 Nm (19%–78% of normal shoulders). External and internal rotation tasks could be performed by only 13 patients (14 shoulders; nine primary, five revisions) generating 9 Nm ± 4 Nm and 8 Nm ± 3 Nm, respectively (13%–71% of normal shoulders). We found moderate correlations between Constant-Murley, DASH and (D)SST (D = Dutch translation) scores and abduction and external rotation.

**Conclusions:**

Patients with a reverse prosthesis had reduced strength when compared with normal values reported in the literature (only 65% of patients could perform the protocol). This effect was greatest for external rotation and might explain clinical outcomes with which a moderately strong relationship was observed. Our observations suggest limited strength is a major factor in reduced ROM.

## Introduction

The reverse shoulder prosthesis is one surgical option for treatment of cuff tear arthropathy and shoulder pseudoparalysis resulting from a massive cuff tear, severe fractures, prosthetic revision, and tumor surgery [5]. Owing to the mechanical advantage of a medialized center of rotation, the reverse shoulder prosthesis offers a potentially valuable surgical option and has become an alternative in situations in which the rotator cuff and/or the proximal humerus are destroyed or absent [5], with a satisfying reduction of pain after surgery [12, 31].

However, because of the wide variation in published values for active elevation after reverse shoulder replacement (ranging from 88° to 138° [3, 36]), the degree to which this prosthesis restores arm strength is not fully defined. Bergmann et al. previously found a better passive than active ROM [3] and presumed that the limited glenohumeral motion of the reverse prosthesis resulted from a lack of joint torque generation rather than a structural limitation caused by the prosthetic design.

Shoulder strength mostly has been evaluated using subjective methods such as traditional manual muscle testing and handheld dynamometry, which mainly focus on isometric muscle strength. The strength measure in the Constant-Murley score [8, 9] is also isometric. Because most functional activities are dynamic, evaluating isokinetic shoulder strength may be more appropriate when relating strength to functional performance and clinical outcome. However, data for isokinetic strength measurements around the shoulder are available only for normal healthy subjects [7], patients after open fixation of glenoid rim fractures [33], open [1, 10] and arthroscopic anterior stabilization [15, 21], rotator cuff surgery [4, 11, 14, 34, 43], with adhesive capsulitis [26, 27, 41], subacromial impingement [16, 24, 30], and pectoralis major muscle rupture [17], but not for patients with a reverse shoulder prosthesis.

Our objective was to perform a pilot study to measure isometric shoulder strength in patients who underwent either a primary or revision reverse shoulder replacement. We asked the following questions: (1) what joint torques can patients with a reverse shoulder prosthesis produce isokinetically, and (2) does this force-generating capacity correlate with functional scores?

## Materials and Methods

Between May 2000 and September 2007, we treated 45 patients (49 shoulders) with a reverse shoulder prosthesis (Tornier®; Edina, MN, USA). Of these, 33 patients (19 women and 14 men), volunteered to participate in this study. Ten patients had surgery on the left side, 19 on the right side, and four on both sides (total of 37 shoulders). In 21 patients, the indication for the reverse prosthesis was cuff tear arthropathy, and in 16 patients the indication was revision after a failed primary placed hemiprosthesis or total shoulder prosthesis. The average time between surgery and measurement was 23 ± 14 months (range, 4–63 months). Mean age of the patients was 72 ± 8 years (range, 58–85 years). The minimum clinical followup was 4 months (mean, 23 months; range, 4–63 months). The institutional ethics committee approved the research protocol and all patients gave their written informed consent before the experiment.

All patients underwent surgery under general anesthesia with an interscalene nerve block in the beach-chair position. We used a standard deltopectoral approach in all patients. All glenoid components had been placed inferior on the glenoid surface with no inferior or superior inclination. Thirty patients had a 36-mm component implanted and seven had a 42-mm sphere diameter. The humeral components had all been placed in 10° to 20° retroversion, and in 32 patients, the teres minor and subscapularis muscles were still intact.

Postoperative management was the same for all patients, consisting of a sling and passive ROM exercises for 6 weeks. After 6 weeks, active assisted ROM exercises were started and at 3 months, strengthening exercises were added to the rehabilitation program.

Shoulder strength was measured with an isokinetic dynamometer, which provides constant velocity with accommodating resistance throughout a joint’s ROM. This resistance is provided using an electric or hydraulic servo-controlled mechanism at a user-defined constant velocity [13]. Two isokinetic protocols were performed to measure the strength of the subjects’ shoulder muscles on the surgically treated side using the Biodex System 3 Pro dynamometer (Biodex Medical Systems, New York, NY, USA). These protocols consisted of an abduction and adduction task and an external and internal rotation task with the arm in 60° abduction in a sitting position with securing bands around the subject’s chest and pelvis. For the abduction and adduction task, the chair was rotated 75° around the vertical axis with the dynamometer in neutral position and 10° tilted. For the external and internal rotation task, the chair was in the neutral position with the dynamometer rotated 20° and 50° tilted. After one session of the abduction and adduction or the external and internal rotation task, the subject had a 60-second recovery time after which the same task was repeated. All tasks were repeated five times at 60° per second with a minimum standard threshold of 15° per second to start the measurements defined by the Biodex System. For each motion, the average maximal torque (Nm) was determined over the two sessions. The subjects were instructed and encouraged to reach the highest possible force level during these tasks. Negative axial rotation was defined as external rotation and positive axial rotation as internal rotation.

Codine et al. [7] reported a systematic review of the literature on isokinetic strength of the shoulder until 2005. We used PubMed to identify other articles providing data for normal shoulder torque values from 2005 and onward [2, 20, 37, 42]. From those studies we took the abduction-adduction and/or external-internal rotation torque values for 60° per second or less and combined those to make comparison possible with our obtained data (Table 1).

**Table 1:** Maximum generated force in Nm for our data compared with the literature at similar or lower velocities

Study	Subject	Mean age (years)	Dominance or side	Velocity	Abduction	Adduction	External rotation	Internal rotation
Ambrosio et al. [2]	M and F wheelchair users	43		60°/second	50.0	42.5	28.2	32.3
Codine et al. [6]	M volunteers	26	D	60°/second			39.8	56.4
			ND				42.7	56.2
	M runners	23	D	60°/second			44.6	57.2
			ND				45.8	54.7
	M tennis players	26	D	60°/second			39.0	57.3
			ND				40.1	54.0
	M baseball players	20	D	60°/second			39.9	65.3
			ND				39.8	55.5
Greenfield et al. [19]	M and F	25		60°/second			14.1	15.4
Harbo et al. [20]	M volunteers	53		60°/second	59.5	83.1		
	F volunteers	52		60°/second	37.4	45.7		
Ivey et al. [22]	M	36		60°/second	37.5	61.0	21.8	33.2
	F	26		60°/second	19.5	34.2	13.0	17.9
Sirota et al. [35]	M baseball players	24	D	60°/second			48.8	51.6
			ND				44.2	52.3
Stickley et al. [37]	F volleyball athletes	13		60°/second			16.8	22.3
Tis and Maxwell [39]	F	25	D	60°/second			24.7	23.3
			ND				24.7	21.2
Verney et al. [42]	M volunteers	73		60°/second	46.0			
				30°/second	50.0			
McMaster et al. [28, 29]	M water polo players	26	Right	30°/second	51.8	99.1	38.2	65.9
			Left		49.4	92.7	34.8	57.8
	M swimming athletes	20	Right	30°/second	48.1	99.1	33.7	66.8
			Left		48.6	102.2	31.8	55.9
	M volunteers	22	Right	30°/second	35.3	54.0	29.1	39.9
			Left		38.2	52.7	28.1	36.8
Otis et al. [32]	M	26	D	48°/second	49.6		26.6	42.2
			ND		46.4		26.6	38.0
Current study	M and F reverse shoulder prosthesis	72		60°/second	15.2	16.1	9.3	8.2

M = male; F = female; D = dominant; ND = nondominant.

For clinical evaluation, we obtained preoperative and postoperative (absolute and relative) Constant-Murley scores [8, 9], postoperative DASH score [23], and the (D)SST [23, 40]. The absolute Constant-Murley score assesses the overall shoulder function and has a maximum score of 100 points. The relative Constant-Murley score is corrected for the age- and sex-related decline in force-generating capacity [46]. It is expressed as a percentage of the respective reference values. The DASH is a 30-item questionnaire that evaluates functional disability in everyday activities, work, and sports. It includes symptoms, physical, social, and psychological function. A DASH score of 0 indicates good shoulder function, or no disability, and the maximum score of 100 indicates no function. The (D)SST is a questionnaire consisting of 13 yes or no questions including subjective items and items that require patients to complete a physical exercise. It evaluates shoulder function in daily activities and a maximum score of 13 indicates good shoulder function.

We used a t-test to determine the difference in mean maximum generated torque at 60° per second between primary and revision cases. For this same group, the effect size was determined by calculating the Cohen’s d. The relationship between the clinical outcome scores (Constant-Murley, DASH, and [D]SST) and strength data was evaluated on the basis of a Pearson product-moment correlation.

## Results

Only 23 patients (24 shoulders; 13 primary and 11 revisions) were able to generate sufficient velocity to perform the test, resulting in a mean abduction torque of 15.2 Nm ± 6.6 Nm for the whole group with no substantially better value for the primary prostheses compared with the revisions (Table 2). For the external and internal rotation torques, these values varied between 13% and 71%. We found similar torque values for adduction also with no major difference between primary and revision cases. The external and internal rotation tasks could be performed by only 13 patients (14 shoulders; nine primaries, five revisions). Mean external rotation torque was 9.3 Nm ± 4.4 Nm for the whole group with no major differences between the primary and revision groups. Internal rotation force tended to be higher (p = 0.07) for primary prostheses with a torque of 8.2 Nm ± 2.6 Nm for the whole group (Table 2). Compared with normal healthy subjects (Table 1), patients with a reverse prosthesis who could generate sufficient force to perform the tasks had abduction and adduction torques of 19% to 78% of those of a normal shoulder at a velocity of 60° per second.

**Table 2:** Mean maximum generated force (Nm) and SD at 60° per second for the whole group

Maximum torque at 60° per second	All shoulders (N = 24)	Primary (N = 13)	Revision (N = 11)	p value primary versus revision	Cohen’s d primary versus revision
Abduction	15.2 ± 6.6	16.3 ± 5.6	13.4 ± 7.6	0.30	0.43
Adduction	16.1 ± 10.0	20.4 ± 11.8	11.8 ± 6.0	0.11	0.92
	(N = 14)	(N = 9)	(N = 5)		
External rotation	9.3 ± 4.4	9.3 ± 4.7	7.9 ± 4.0	0.58	0.32
Internal rotation	8.2 ± 2.6	9.2 ± 2.1	6.0 ± 2.5	0.07	1.38

**Fig. 1:**
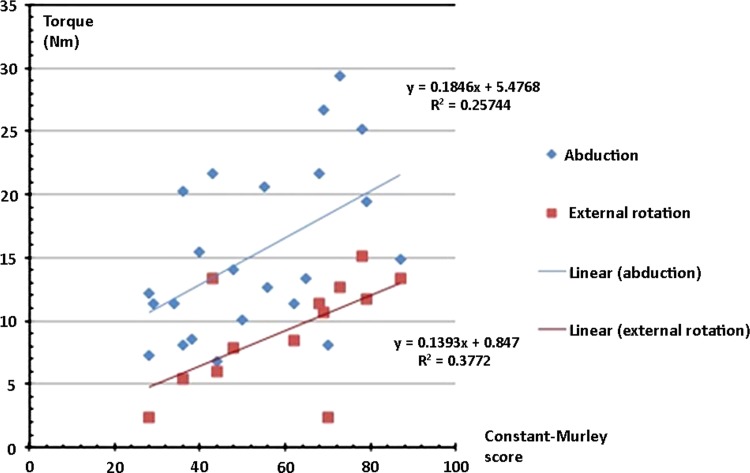
Pearson’s correlation between the maximal abduction and external rotation torque at 60° per second and the postoperative Constant-Murley score show a correlation between the force-generating capacity of patients with a reverse shoulder prosthesis and their postoperative Constant-Murley score for abduction and external rotation.

We found a correlation between the postoperative Constant-Murley score and the abduction and external rotation torques (Fig. 1). Similar correlations were found for the DASH score and (D)SST (Table 3), with the maximum torque values at 60° per second. There was no major correlation for the adduction and internal rotation motions. An overview of all the clinical outcome scores of the whole group, the primary and the revision cases is presented (Table 4).

**Table 3:** Pearson’s correlation between maximum torque at 60° per second and the postoperative Constant-Murley score, DASH, and (D)SST

Maximum torque at 60° per second	Constant-Murley score	DASH	(D)SST
Abduction	0.507 p = 0.014	−0.572 p = 0.004	0.519 p = 0.011
Adduction	0.393 p = 0.183	−0.319 p = 0.29	0.408 p = 0.166
External rotation	0.614 p = 0.026	−0.531 p = 0.062	0.600 p = 0.03
Internal rotation	0.441 p = 0.216	−0.498 p = 0.205	0.455 p = 0.206

(D)SST = Dutch translation of the Simple Shoulder Test.

**Table 4:** Constant-Murley scores, relative Constant-Murley scores, DASH scores, and (D)SST

Scores	All shoulders ± SD (range)	Primary ± SD (range)	Revision ± SD (range)
Constant-Murley preoperative	24 ± 11 (5–47)	28 ± 9 (13–47)	20 ± 12 (5–47)
Constant-Murley postoperative	50 ± 21 (8–87)	59 ± 20 (8–87)	38 ± 18 (11–73)
Relative Constant-Murley preoperative	33% ± 17% (7–71)	38% ± 14% (19–68)	27% ± 18% (7–71)
Relative Constant-Murley postoperative	70% ± 31% (9–124)	83% ± 30% (9–124)	53% ± 22% (14–92)
DASH postoperative	43.9 ± 25.6 (1.7–84.2)	31.5 ± 24.4 (1.7–77.5)	60.3 ± 17.1 (31.2–84.2)
(D)SST postoperative	7 ± 4 (0–13)	8 ± 4 (0–13)	4 ± 3 (1–10)

(D)SST = Dutch translation of the Simple Shoulder Test.

## Discussion

The reverse shoulder prosthesis provides a surgical option for conditions such as cuff tear arthropathy, shoulder pseudoparalysis resulting from massive cuff tear, severe fractures, prosthetic revision, and tumor surgery [5] with generally satisfying postoperative results [12, 31]. However, the contribution of this prosthesis to restoration of arm function is less clear. Previous research [3] suggests the limited glenohumeral motion of the reverse prosthesis seems to be the result of a lack of joint torque generation rather than a structural limitation caused by the prosthetic design. Therefore, the evaluation of isokinetic shoulder strength after reverse shoulder replacement may be of interest in modeling dynamic upper extremity function, particularly where comparative data are not currently available for this clinical scenario. We therefore (1) determined joint torques in patients with a reverse shoulder prosthesis and (2) determined whether force-generating capacity correlates with functional scores.

We note limitations to our study, one of which is the absence of proper control data. First, ideally a comparison would be made with an age-matched control group without cuff disorders. However, with a prevalence of 31% of asymptomatic (ie, unrecognized) cuff tears in individuals between 70 and 79 years old [38] and a prevalence of 51% in individuals older than 80 years [38], this is not feasible without extensive screening. Another possibility would be to compare the outcomes with those of the contralateral side in the same patient. However, cuff disorders in the contralateral shoulder are not uncommon, as reported by Yamaguchi et al. [45] in their demographic and morphologic study of rotator cuff disease. The average age for patients with a bilateral cuff tear in their group was 67.8 years and logistic regression analysis indicated a 50% likelihood of a bilateral tear after the age of 66 years. Furthermore, patients with a full-thickness symptomatic tear had a 35.5% prevalence of a full-thickness tear on the contralateral side. In our patient population, 12% already had a reverse prosthesis on both sides, showing that our patient group was not suitable to use the contralateral side as a comparison. As a consequence data had to be compared with norm data from the literature. Second, we had a broad range of followup times for the force measurements and clinical outcome scores. Ideally the measurements should have been performed at the same time postoperatively for every patient with a minimum followup of 1 year. This is also true for the clinical outcome scores, because they require time to stabilize. In the scope of this study, it was not possible to include patients with the same followup period, as this would have required an inclusion period of several years. We therefore chose to include all patients available from our pool of treated patients, which inevitably led to a large range in followup times. It is not certain what effect the followup time will have had, which especially applies for the elderly population for whom recovery might be counteracted by ageing effects. Given the number of available patients, controlling for age and followup will be virtually impossible whereas including larger groups and testing for those factors also do not seem to be realistic options. Third, we limited our measurements to 60° per second. Isokinetic strength measurements have been performed at different velocities, mostly from 60° per second to 300° per second [7] with some exceptions at 30° per second (Table 1). In these measurements, the applied torque needs to increase above a threshold value to successfully perform a certain task at higher speeds. Because the physiologic changes at older age lead to a decline in force-generating capacity and the reverse shoulder prosthesis is implanted mainly in patients with a mean age of 72 years [18, 36], similar to the average age of the participants in our study, we decided to apply a relatively low velocity of 60° per second. Considering our data and the number of patients unable to perform the tests (Table 2), it appeared that even 60° per second was too high for most of the patients with a reverse prosthesis. Future research investigating force production of this patient population should incorporate velocities less than 60° per second. Whether a lower velocity would lead to substantially more successful tests however is unknown; in our protocol a standard threshold of 15° per second was used to start measurements, which even proved to be too much for some of our patients.

Trying to place our obtained torque values (Table 2) in perspective, we compared our data with those of normal healthy subjects (Table 1). From this comparison we can conclude that patients with a reverse prosthesis who can generate sufficient force to perform the tasks have abduction and adduction torques of 19% to 78% of those of a normal shoulder at a velocity of 60° per second. For the external and internal rotation torques, these values vary between 13% and 71%. However, those normal values were based on younger subjects than our group of patients and in most series they include groups of athletes. If we compare our data with the only age-related series of Verney et al. [42], our patients have an abduction torque of 33% of that of 10 male elderly volunteers. It is not clear what causes this relatively low abduction torque. From the total of 37 shoulders, only 23 patients (24 shoulders) could generate enough force to perform the abduction and adduction tasks and for the external and internal rotation, the number of patients was even less (Table 2). The difference between those two tasks can be explained by the changed biomechanics caused by the reverse shoulder prosthesis. By displacing the center of rotation medially, more fibers of the anterior and posterior parts of the deltoid muscle are recruited for anteflexion or abduction of the arm and therefore fewer fibers are available to internally or externally rotate the arm [5]. Our study group included patients with primary and revision implantations. In revision surgery with a reverse prosthesis, the improvement of function is reportedly only to approximately 70° of active elevation [25], with a higher complication rate [44] than with primary surgery. Therefore, we expected to find a difference in force-generating capacity between the two groups (Table 2) in favor of the primary prosthesis. However, this could not be confirmed for the abduction and adduction tasks because 62% of the primary and 69% of the revision cases were able to generate enough force. For the internal and external rotation tasks, it was 43% and 31% respectively, confirming our expectation and explained by the previously mentioned change of biomechanics after a reverse prosthesis.

Impaired shoulder strength is likely one of the causes of active ROM limitations. The correlations we found between clinical outcome scores (Constant-Murley, DASH, and [D]SST) and the abduction and external rotation torque values (Table 3) support this contention. Functional outcome probably is not determined by simple ROM ranges alone, but also by the actual capacity for material handling in elevated and axially rotated arm positions. For example, it can be expected that patients who have good anteflexion or abduction with limited external rotation strength define their functional outcome as poor. Therefore, it seems logical that greater external rotation torque provides a better functional outcome. Although our findings support this notion, only 13 of a total of 37 shoulders actually were able to generate enough force to perform the tasks at 60° per second. Testing under lighter conditions (30° per second) could have provided more data but probably would not have led to another observation.

Patients with a reverse prosthesis were moderately to strongly limited in strength, which was the case for abduction and adduction and even more for external and internal rotation. However, future isokinetic data collection in these patients should be performed at a lower velocity than 60° per second. Results for strength correlated with clinical outcome scores (Constant-Murley, DASH, and [D]SST) indicating moderately strong relationships and a moderate predictive value of the outcome scores. Although it is likely that lower isokinetic shoulder strength in patients with joint arthroplasties is a major factor in reduced ROM, the actual causes of loss of strength would need to be identified in future studies.
